# Vascular dysfunction and increased metastasis of B16F10 melanomas in *Shb* deficient mice as compared with their wild type counterparts

**DOI:** 10.1186/s12885-015-1269-y

**Published:** 2015-04-08

**Authors:** Guangxiang Zang, Karin Gustafsson, Maria Jamalpour, JongWook Hong, Guillem Genové, Michael Welsh

**Affiliations:** 1 Department of Medical Cell Biology, Uppsala University, Box 571, Husargatan 3, 75123 Uppsala, Sweden; 2 Department of Medical Biochemistry and Biophysics, Division of Vascular Biology, Karolinska Institutet, Stockholm, Sweden; 3 Present address: Department of Medical Bioscience, Umeå University, Umeå, Sweden

**Keywords:** Shb, Melanoma, Vascular permeability, Metastasis, CD8+ cells, Pericytes

## Abstract

**Background:**

Shb is a signaling protein downstream of vascular endothelial growth factor receptor-2 and *Shb* deficiency has been found to restrict tumor angiogenesis. The present study was performed in order to assess metastasis in *Shb* deficiency using B16F10 melanoma cells.

**Methods:**

B16F10 melanoma cells were inoculated subcutaneously on wild type or *Shb* +/− mice. Primary tumors were resected and lung metastasis determined after tumor relapse. Lung metastasis was also assessed after bone marrow transplantation of wild type bone marrow to *Shb* +/− recipients and *Shb* +/− bone marrow to wild type recipients. Primary tumors were subject to immunofluorescence staining for CD31, VE-cadherin, desmin and CD8, RNA isolation and isolation of vascular fragments for further RNA isolation. RNA was used for real-time RT-PCR and microarray analysis.

**Results:**

Numbers of lung metastases were increased in *Shb* +/− or −/− mice and this coincided with reduced pericyte coverage and increased vascular permeability. Gene expression profiling of vascular fragments isolated from primary tumors and total tumor RNA revealed decreased expression of different markers for cytotoxic T cells in tumors grown on *Shb* +/− mice, suggesting that vascular aberrations caused altered immune responses.

**Conclusions:**

It is concluded that a unique combinatorial response of increased vascular permeability and reduced recruitment of cytotoxic CD8+ cells occurs as a consequence of *Shb* deficiency in B16F10 melanomas. These changes may promote tumor cell intravasation and metastasis.

**Electronic supplementary material:**

The online version of this article (doi:10.1186/s12885-015-1269-y) contains supplementary material, which is available to authorized users.

## Background

Angiogenesis inhibition [1] has become a clinically accepted treatment of numerous malignant diseases (www.cancer.gov/cancertopics/factsheet/Therapy/angiogenesis-inhibitors). The primary targets of anti-angiogenic therapy so far have been the main angiogenic factor VEGF (vascular endothelial growth factor) or its receptor VEGFR-2 [2,3]. Despite these advances, this therapeutic regimen is clearly not as straightforward as initially thought and certain reports suggest that some tumors may become more aggressive showing increased invasiveness and metastatic spread as a consequence of anti-angiogenic treatment [4,5].

SHB (Src homology domain containing protein B) is an adaptor protein [6] operating downstream of VEGFR-2 [7], the receptor active in VEGF’s angiogenic response [8]. The *Shb* knockout mouse phenotype was found to be pleiotropic with aberrations in female reproduction [9,10], glucose homeostasis [11], the T lymphocyte response to T cell receptor stimulation [12,13] and the vasculature [14-16]. In particular, the vasculature displayed reduced angiogenesis and vascular permeability in response to VEGF [14,16]. Consequently, absence of one *Shb*-allele conferred a restriction on tumor growth of Lewis lung carcinoma and T241 fibrosarcoma cells [14] and of inheritable RT2 (rat insulin promoter-SV 40 T antigen) insulinomas [15]. In addition to the ameliorating effects of *Shb*-deficiency on pathological angiogenesis, *Shb* knockout mice displayed vascular abnormalities that resulted in impaired recovery after ischemic injury [16]. *Shb* knockout endothelial cells show reduced responsiveness to VEGF-stimulation with respect to ERK (extracellular-signal regulated kinase), Akt, FAK (focal adhesion kinase), Rac1 and myosin light chain kinase [14,17]. In concert, this abnormal signaling signature affects endothelial cell migration and adherens junction dissolution in response to VEGF [14,16,17], explaining the vascular dysfunction *in vivo*.

Melanomas are highly invasive cancers that metastasize at an early stage [18,19]. Since *Shb*-deficiency appears to reduce tumor growth by restricting the angiogenic expansion of the vasculature, the question of whether this will cause increased tumor invasiveness and metastasis or not remains unanswered. In the RT2 insulinoma model, no evidence for increased liver metastasis was obtained [15]. However, melanomas have a high inherent propensity for metastasis and for that reason, B16F10 melanoma cells were grown in *Shb*-deficient mice and the numbers of lung metastases determined. Indeed, it was observed that melanoma metastasis was increased in *Shb*-deficient mice because of a defective vasculature showing elevated vascular permeability and diminished recruitment of CD8+ cells to vascular structures.

## Methods

### Animals

*Shb* +/+ and +/− mice were bred on the C57Bl6 background for 8 generations (F8). Alternatively, *Shb* −/− and +/+ mice bred for four generations (F4) on that strain of mice were used. It was previously shown that *Shb* −/− mice cannot be obtained after breeding for more than 4 generations onto the C57Bl6 background [10]. All animal experiments had been approved by the local animal ethics committee at the Uppsala County Court.

### Tumor cell injections

B16F10 melanoma cells (2 x 10^5^) were injected subcutaneously in the subscapular region. When the tumor reached a size of 0.5 – 1 cm^3^ (determined by a caliper) the tumor was resected under anesthesia. Excised tumors were weighed for size determination. The mice were housed for an additional 10–19 days (commonly, but not always, there was a tumor relapse deciding the end-point of the experiment) after which the mice were sacrificed. Some of the mice were injected with 2 mg/kg FITC-conjugated Dextran-70000 (46945, Sigma, St. Louis, MO, USA) 30 minutes before sacrifice into the tail vein in order to determine blood vessel permeability. For lung seeding, 200000 B16F10 cells were injected in the tail vein and the mice maintained for three weeks before sacrifice. Lungs were excised and macroscopically visible metastases counted. The area was also inspected carefully for lymph node metastases but none were detected. The resected primary tumor was frozen on dry ice for immunofluorescence staining or stored in RNA-later (Quiagen, Hilden, Germany) for subsequent RNA preparation.

### Immunofluorescence

Excised tumors were sectioned (5 μm) and subjected to immunofluorescence staining for CD31 (553370, BD Pharmingen, Franklin Lakes, NJ, USA), VE-cadherin (vascular endothelial-cadherin) (AF1002, R&D Systems, Minneapolis, MN, USA), desmin (ab6322, Abcam, Cambridge, UK) and fibrin/fibrinogen (GAM/Fbg/7S, Nordic Immunological Laboratories, Eindhoven, the Netherlands) as previously described [15].

At least five pictures were taken randomly of each tumor using a Nikon fluorescence and confocal C-1 microscope (Nikon, Japan). The area, diameter, perimeter of blood vessels, the fibrin spread area and pericyte covered length were measured with Image J software. Quantification of blood vessel permeability of FITC-conjugated Dextran was performed using Photoshop software.

### Isolation of vascular fragments

Microvascular fragments were isolated from B16F10 melanomas grown on *Shb* +/− and control mice as previously described [20]. Briefly, tumors (0.5-1.0 cm^3^) were perfused with Hanks’ salt solution under anesthesia and then excised. They were then cut into small pieces and digested in 1.5 ml of 5 mg/ml Collagenase A (#103586, Roche Diagnostics, Basel, Switzerland) and 100 U/ml DNaseI (Invitrogen, Carlsbad, CA) Hanks’ solution per tumor for 15 min at 37°C. The tumor suspension was pipetted, filtered through a 70 μm diameter cell strainer (BD Bioscience, Franklin Lakes, NJ), washed and filtered a second time with a 40 μm cell strainer. After washing, the cells were incubated with CD31-coated Dynabeads. The magnetic beads (with the captured vascular fragments) were collected using a magnetic rack, washed extensively after which RNA was prepared from the captured cells using the Quiagen RNeasy Mini Kit (Quiagen, Hilden, Germany). Endothelial cells were isolated as described [14].

### Gene expression

Total RNA of tumor was extracted according to RNeasy mini kit (74104; Qiagen) with RNase-Free DNase set (79254,Qiagen). One-step quantitative real-time RT-PCR was performed with QuantiTect™ SYBR®Green RT-PCR-kit (204243,Qiagen) on a LightCycler™ real-time PCR machine (lightcycler 2.0; Roche, Mannheim, Germany). Cycle threshold (Ct) values were determined with the LightCycler Software v3.5 (Qiagen). Gene expression was normalized for differences in RNA by subtracting the corresponding β-actin Ct-value. Statistical comparisons were made on normalized Ct-values. TaqMan qPCR gene expression analysis (Taqman, Life technologies, Carlsbad, CA) was used for analysis of PDGF-D (platelet-derived growth factor), CSFR2 (colony stimulating factor receptor 2), CXCL12 (chemokine C-X-C motif ligand), CXCR-4 (CXCL receptor) and CXCR-7.

### Microarray analysis

High quality vascular fragment RNA from five tumors of each genotype was analyzed with Affymetrix 1.0 ST chips at the microarray core facility at Uppsala University Hospital (Uppsala Array Platform, Department of Medical Science, Science for Life Laboratory, Uppsala University Hospital, Sweden). Ingenuity software (Quiagen, Hilden, Germany) was used to perform pathway analysis on the microarray samples.

### Bone marrow transplantation for generating chimeric mice

Iliac bones, femurs and tibias were collected from 8 to 10 week-old C57Bl/6 *Shb* +/+ and *Shb* +/− donor mice and processed as described [21,22]. Cell numbers were determined and 1.5 x10^6^ cells were transplanted into congenic *Shb* +/+ or *Shb* +/− recipients by retro-orbital injection. Prior to the bone marrow transfer, the recipients were irradiated [22] with a split dose separated by two hours of 10 Gy. Peripheral blood chimerism in the recipient mice was determined 6 weeks post-transplantation by bleeding 100–200 μl blood in 0.05 mM EDTA. Total RNA was isolated from the remaining leukocytes with the RNeasy mini kit (74104; Qiagen). The *Shb* gene expression in the chimeric mice was compared between different *Shb* genotypes, following the real-time RT-PCR procedure.

In separate bone marrow transplantation experiments we assessed chimerism after bone marrow transfer by CD45.1 and CD45.2 staining after transfer of CD45.2-positive bone marrow to CD45.1-positive recipients. In such experiments, the donor bone marrow repopulated the host with more than 75% efficiency (results not shown).

### Statistics

All values are given as means ± SEM. Probabilities (P) of chance differences between the groups were calculated with Mann–Whitney rank sum test (tumor metastases) or Students’ t-test (all other comparisons). Relative frequencies of metastasis were determined as follows: each mouse was categorized with category 0 having 0 metastases, category 1 having 1–5 metastases and category 2 having >5 metastases.

## Results

### B16F10 melanoma growth and metastasis in *Shb* +/− mice

**Figure 1:**
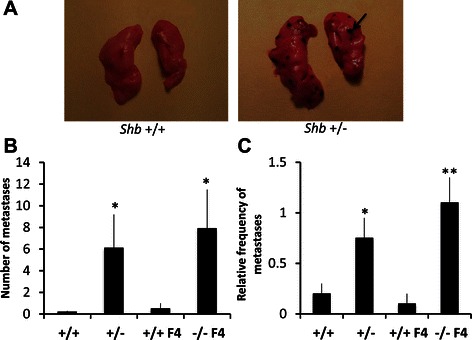
**B16F10 melanoma lung metastasis in*Shb*+/+, +/− and −/− mice. (A)** Pictures show examples of lungs without (+/+) and with (+/−) B16F10 melanoma metastases. **(B)** At time of sacrifice, numbers of lung metastases were determined (arrow indicates a metastasis). These are given as absolute numbers for each mouse **(B)** or scored as relative frequencies after grouping in categories **(C)** (0 = 0, 1 = 1–5, 2= >5). Means ± SEM for n = 16 +/+, n = 16 +/−, n = 11 +/+ F4 and n = 11 −/− F4. * and ** indicate p < 0.05 and p < 0.01, respectively when compared with corresponding +/+ controls by Mann–Whitney U test.

Tumor growth, vasculature and metastasis was investigated using the B16F10 melanoma model [23]. In Additional file 1: Figure S1, tumor growth in *Shb* +/+ and *Shb* +/− mice is shown. No statistically significant difference was observed although 5 of 18 +/− tumors were much larger than the others on day 14 thus explaining the prominent error bar. Tumor resection was followed by a time period before animal sacrifice that occurred at the same time point for both groups of mice (Additional file 1: Figure S1). When determining lung metastases at sacrifice, the *Shb* +/− mice showed an increased number of lung metastases compared with controls (Figure 1A,B). Lung metastases were also categorized as zero (0 metastases), low = 1 (1–5 metastases) and high = 2 (>5 metastases) and using this scoring method, the *Shb* +/− mice also exhibited an increased frequency of metastasis (Figure 1C).

*Shb* −/− mice can be obtained after breeding for maximally four generations onto the C57Bl6 background (F4) and we thus determined numbers and relative frequencies of metastasis in F4 *Shb* −/− mice, comparing them with corresponding F4 wild type controls (Figure 1B, C). Similar numbers were obtained as was seen in the F8 *Shb* +/− mice showing an increased absolute number of metastases, regardless of whether determined as numbers or relative frequency of metastasis when scored in categories (Figure 1B, C). For the following experiments, *Shb* +/− F8 mice were studied and compared with F8 +/+ controls since F4 mice are not considered inbred.

### Lung seeding of tail vein-injected B16F10 melanoma cells

B16F10 melanoma cells were also injected in the tail vein to assess metastatic lung seeding of the injected cells (Additional file 2: Figure S2). No difference could be noted between wild type or *Shb*-deficient mice after this procedure, indicating that the ability of the target organ to seed tumor cells spread in the bloodstream was not dependent on genotype.

### *Shb* +/− B16F10 melanoma vasculature

**Figure 2:**
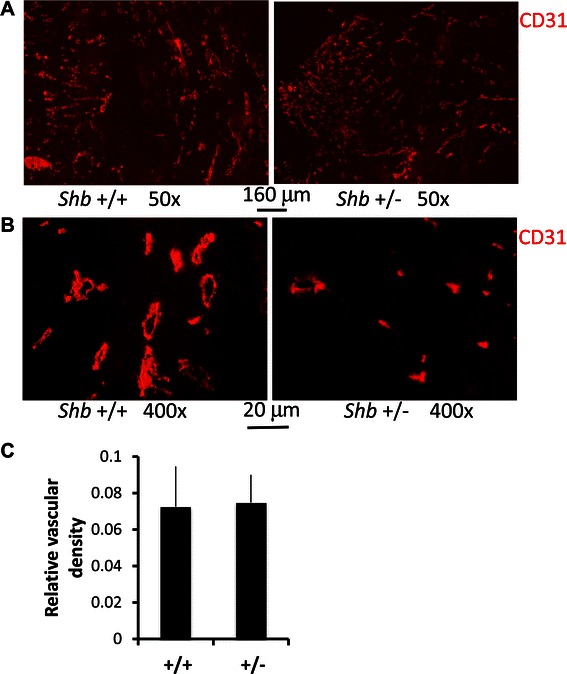
**Vascular characteristics of*Shb*+/+ and +/− B16F10 tumors.** Sections from B16F10 melanomas grown in *Shb* +/+ and +/− mice were stained for CD31 (red, **A**, **B**). Representative images at the magnifications indicated are shown **(A-B)**. Relative vascular density (**C**, grid intersections over a red structure divided by total intersections) were quantitated and given as means ± SEM for 5 tumors each genotype and the value of each tumor was based on five images.

In previous studies, absence of one *Shb* allele conferred reduced tumor angiogenesis [14,15]. We currently decided to investigate the vasculature of B16F10 melanomas grown on the *Shb* +/− background. Surprisingly, no difference in vascular density was observed between *Shb* +/+ and +/− tumors (Figure 2A-C). The vasculature was unevenly distributed in both genotypes with certain areas showing a high vascular density and others not.

### *Shb* +/− melanoma vascular pericyte coverage and permeability

**Figure 3:**
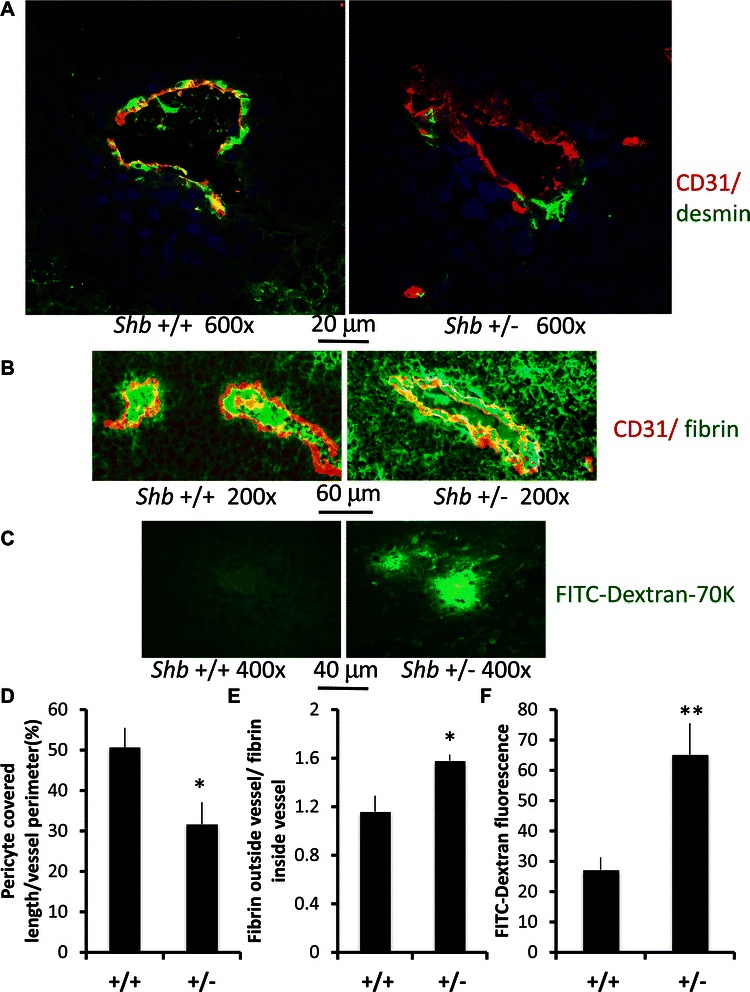
**Pericyte coverage and leakage of fibrinogen in B16F10 tumors grown in*Shb*+/+ and +/− mice.** Sections from B16F10 melanomas were stained for CD31 (red) + desmin (green) **(A)** or CD31 (red) + fibrin (green) **(B)**. Representative images are shown at the magnifications indicated. FITC-Dextran-70 K leakage was also determined by direct immunofluorescence of frozen sections of tumors after injection of the fluorescent dye **(C)**. The percentage vessel coverage by pericytes was quantitated **(D)**, as well as fibrin staining outside the CD31-lines vessel divided by the corresponding value contained within the CD31-positive structure **(E)**. FITC-Dextran fluorescence was determined by Photoshop and given as relative values **(F)**. Means ± SEM are given and five tumors each genotype were analyzed. The value of each tumor was based on five images. * and ** indicate p < 0.05 and 0.01, respectively, by Students’ t-test.

Endothelial pericyte coverage and vascular permeability have been suggested as factors contributing to increased tumor metastasis [24]. Although no apparent difference in the endothelial pericyte coverage of tumors grown in *Shb* deficient mice was previously detected [14], morphological aberrations were noted [15]. These could relate to the abnormal endothelial cell morphology but could also reflect cell-autonomous changes of pericytes as well. We currently set out to investigate the status of pericytes in the *Shb* +/− melanomas by staining for the pericyte marker desmin (Figure 3A, D). We observed reduced pericyte coverage in melanomas grown in *Shb* +/− mice. This reduction correlated with increased fibrin deposits outside the vessel itself (Figure 3B, E), indicating increased vascular leakage. Increased vascular leakage was also noted in melanomas grown in *Shb* +/− mice by assessing FITC-Dextran-70000 fluorescence after injection (Figure 3C, F). Thus the combined data in Figures 2 and 3 provide evidence for a vascular phenotype showing increased vascular permeability that is supportive of B16 melanoma metastasis by allowing melanoma cell intravasation and dissemination.

### Gene expression in vascular fragments isolated from tumors

To understand the molecular mechanisms responsible for the observed vascular abnormalities, vascular fragments were isolated from tumors grown on *Shb* +/+ and +/− mice. Real-time RT-PCR for various angiogenic factors, receptors and chemokines of potential relevance for the vascular phenotype showed no changes in expression of most of these (Table 1), *i.e.* VEGFA, PDGF-AA, PDGF-BB, PDGF-CC, PDGF-DD, angiopoietin-1, angiopoietin-2, VEGFR1, VEGFR2, Tie-2, PDGFRB, GM-CSF, CXCL-12, CXCR-4 and CXCR-7 were unchanged. PlGF (placental growth factor) was increased and PDGFRA was reduced in *Shb* +/− vascular fragments. The decrease in PDGFRA expression, together with decreased vascular coverage of desmin staining, point towards reduction of the B-type pericyte [25] characterized by expression of these markers.

**Table 1:** **Gene expression in vascular fragments**

Gene product	*Shb*+/+	*Shb*+/−
VEGFA	1.0 ± 0.12	1.1 ± 0.15
PlGF	1.0 ± 0.25	1.8 ± 0.22 *
PDGF-AA	1.0 ± 0.15	1.2 ± 0.11
PDGF-BB	1.0 ± 0.21	1.2 ± 0.32
PDGF-CC	1.0 ± 0.09	1.0 ± 0.08
PDGF-DD	1.0 ± 0.64	1.3 ± 0.22
Angiopoietin-1	1.0 ± 0.13	1.1 ± 0.25
Angiopoietin-2	1.0 ± 0.22	1.0 ± 0.34
VEGFR1	1.0 ± 0.16	1.0 ± 0.28
VEGFR2	1.0 ± 0.18	1.1 ± 0.27
Tie-2	1.0 ± 0.22	1.2 ± 0.31
PDGFRA	1.0 ± 0.11	0.7 ± 0.09 *
PDGFRB	1.0 ± 0.27	1.0 ± 0.30
GM-CSF	1.0 ± 0.11	0.8 ± 0.40
CXCL-12	1.0 ± 0.59	0.9 ± 0.22
CXCR-4	1.0 ± 0.46	1.3 ± 0.22
CXCR-7	1.0 ± 0.63	1.4 ± 0.36

RNA isolated from vascular fragments was analyzed by real-time RT-PCR. Ct values were determined using the Roche Lightcycler software and beta-actin values were subtracted to normalize for differences in total amounts of RNA. Relative values shown in table were calculated by the formula 2^-ΔCt (Shb+/− minus Shb+/+)^. *indicates p < 0.05 when compared with wild type. N = 5–7.

These findings were further supplemented with microarray analysis using Affymetrix 1.0/2.0 chips (Additional file 3: Table S1). The most striking findings were reduced expression of IL-6 (interleukin) and numerous markers for active cytotoxic CD8+ cells. These include several granzymes (*Gzmc*, *Gzmd*, *Gzme*, *Gzmf* and *Gzmg*), *Pdcd1l2* (PD-L2) and *Xcl1*. One gene that showed increased expression in *Shb* +/− vascular fragments was *Pten*, which exerts an antagonistic role relative that of phosphatidylinositol 3-kinase. The analysis of the microarrays by Ingenuity software revealed that cell-to-cell signaling and interaction, inflammatory response, immune cell trafficking, cell-mediated immune response, immunological disease, and natural killer cell proliferation and development were amongst the most deregulated pathways under the “top diseases and biological functions” heading. Ingenuity analysis also confirmed that granzymes and IL-6 were significantly downregulated in the *Shb +/−* microvascular fragments. Collectively, microarray data points to a decrease in the recruitment of immune cells, particularly CD8+ cells, to the *Shb +/−* tumors as compared to wild type controls.

**Figure 4:**
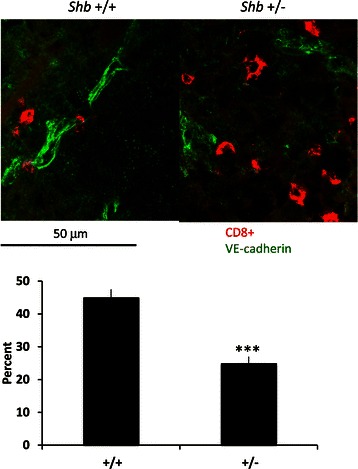
**Proximity of CD8+ T cells with vascular structures.** Cryosections of subcutaneous B16F10 melanomas were stained for CD8+ (red) and VE-cadherin (green). Pictures were taken at 20X (for statistical analysis) or 40X (for representative image) original magnification and the numbers of CD8+ cells immediately adjacent (direct contact) to VE-cadherin positive cells were scored in percent of total CD8+ cells for each host genotype present in the images. Representative images and statistics are shown as percent adjacent CD8+ cells (means ± SEM) for 4 tumors each genotype (5 images each tumor).

The possible involvement of CD8+ cells in affecting rates of metastasis was studied additionally by tumor staining for CD8a. Tumor numbers of CD8+ cells was difficult to assess due very uneven distribution of these cells within the tumors. However, the association of CD8+ cells with vascular structures could be quantified by double staining for CD8a and the vascular marker VE-cadherin showing a lower percentage CD8+ cells in direct contact with endothelial cells in *Shb* +/− tumors (Figure 4).

**Figure 5:**
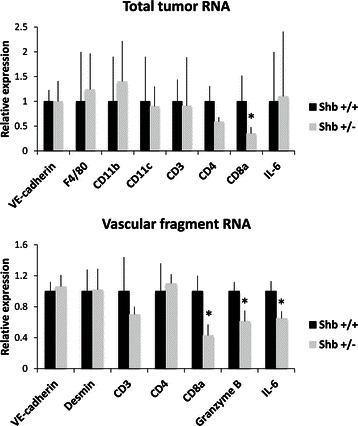
**Gene expression in whole tumors and isolated vascular fragments.** Total tumor RNA or RNA from isolated vascular fragments were isolated from either host genotype and analyzed by real-time RT-PCR. Ct-values were determined using the Roche Lightcycler software and beta-actin values were subtracted to normalize for differences in total amounts of RNA. Relative values shown in table were calculated by the formula 2^-ΔCt (Shb+/− minus Shb+/+)^. *indicates p < 0.05 when compared with wild type. N = 5–7.

To obtain reliable estimates of tumor CD8+ cell infiltration, CD8a gene expression was determined by real-time RT-PCR of total tumor RNA and was found to be selectively decreased in *Shb* +/− tumors (Figure 5). IL-6 expression was similar in wild type and *Shb* +/− total tumor RNA. In vascular fragments, expression of CD8a as well as granzyme B and IL-6 were decreased as a consequence of *Shb* deficiency. Gene expression analysis of isolated endothelial cells showed significantly less IL-6 gene expression (<1%) compared with that of the vascular fragments, suggesting that passenger lymphocytes provide the main source of IL-6 mRNA in the vascular fragment preparations (results not shown).

### Bone marrow transplantation experiments

**Figure 6:**
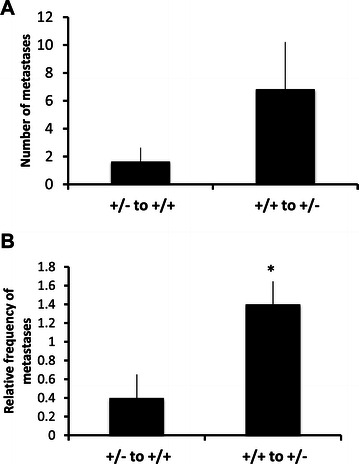
**Effects of bone marrow genotype on B16F10 metastasis.***Shb* +/− mice received +/+ bone marrow and *Shb* +/+ mice received +/− bone marrow after transplantation to recipients that had been depleted of their endogenous bone marrow by irradiation. After 3 months when hematopoiesis had been restored, B16F10 tumors were gown subcutaneously, resected and the mice sacrificed when tumor recurrence occurred (average 34 ± 2 days after initial injection of cells in the +/+ recipient group and 36 ± 0 days in the +/− recipient group). Numbers of lung metastases were then scored and given as absolute values **(A)** or as frequencies of categories **(B)** where 0 = 0, 1 = 1–5 and 2= >5 metastases. Means ± SEM for 5 mice in each group are given. *indicates p < 0.05 by Students’ t-test.

The possibility that immune cells could be responsible for the increased rate of metastasis motivated us to perform bone marrow transplantations and to follow the effect of the hematopoietic cell genotype on metastasis. Wild type bone marrow was transplanted to *Shb* +/− recipients and *Shb* +/− bone marrow was transplanted to wild type recipients. After restitution of hematopoiesis (>3 months) B16F10 melanoma cells were inoculated subcutaneously, primary tumors removed and the appearance of lung metastasis monitored. There was as in Additional file 1: Figure S1 no difference in primary tumor growth, time of resection and time point of final sacrifice between the two groups (results not shown). However, number of lung metastasis was higher (Figure 6) in the *Shb* +/− recipient group receiving wild type bone marrow when scored as categories (0, 1 and 2 as in Figure 1), suggesting that the main determinant responsible for the increased metastasis in *Shb* +/− mice is the vascular genotype and not that of hematopoietic cells.

## Discussion

The current study elucidates the metastatic properties of melanoma cells in relation to the absence of the *Shb* gene. This cancer has been extensively investigated and tumor progression follows a characteristic pattern [19]. The driver mutations in melanomas have recently been mapped [26] and revealed a limited number of genetic changes in this cancer. Melanoma growth is dependent on angiogenesis [18,27,28] and numerous angiogenic factors have been linked to melanoma growth [29,30] although the role of VEGF appears modest [31]. Alternative candidates for support of melanoma growth and metastasis are inflammatory cytokines and chemokines [32]. Factors of particular importance in this context are IL-8 [29], CCL19/21 [33], CXCL12 [34], IL-6 and macrophage migration inhibitory factor [35,36]. The combined data suggest a scenario that utilizes multiple factors for melanoma growth and angiogenesis.

The B16F10 melanoma cell line is a useful model for studying melanoma metastasis *in vivo* [23] and consequently, we tested tumor metastasis in *Shb* deficient mice. The increased rate of lung metastasis observed in *Shb* deficient mice may have several explanations. One possibility is increased vascular permeability due to reduced pericyte coverage. However, gene expression profiling showed less expression of various markers for cytotoxic CD8+ lymphocytes in the *Shb* +/− vasculature and this may offer an alternative suggestion. Due to some yet unknown feature of the *Shb* deficient vasculature, passage of CD8+ cells over the vascular barrier into the tumor is specifically hampered thus causing less CD8+ cell infiltration into the tumor. This may contribute to increased metastasis since it is well established that CD8+ cytotoxic T cells combat melanoma growth and metastasis [37-39]. Indeed, treatment with an activator of CD8+ T cells, Ipilimumab (anti-CTLA-4), together with an inhibitor of VEGF signaling (Bevacizumab) causes perivascular CD8+ cell accumulation [40], thus confirming the relevance of the vasculature for tumor-infiltration of cytotoxic T cells. The bone marrow transplantation experiments further support this notion, since the metastatic phenotype followed the recipient genotype, *i.e.* more metastasis was seen in *Shb* +/− recipients with a *Shb* deficient vasculature despite these having a wild type bone marrow producing wild type blood cells. The decrease in tumor infiltration of CD8+ cells was indeed surprising, considering that vascular permeability was increased these conditions. Apparently, specific mechanisms operating in the vascular component control lymphocyte endothelial transmigration. Lymphocyte extravasation depends on numerous endothelial processes but selective mechanisms operating for lymphocytes and in particular for CD8+ lymphocytes are poorly defined.

The microarray analysis provides no obvious explanation for leaky vascular phenotype, although changes in the expression of numerous cytokines/chemokines could contribute to this. PlGF is one factor that has been suggested to increase vascular permeability [41-43] but more recent studies suggest a modest role, if any, of PlGF for vascular permeability [44,45]. Reduced PDGFRA expression could be explained as downregulation of its expression in a subset of pericytes, the B-pericyte [25]. Apart from the reduction in PDGFRA expression, our study shows decreased perivascular desmin staining, a feature shared by these B-pericytes. Alternatively, reduced PDGFRA expression might indicate a reduction in myofibrillar cells. PDGFRA-expressing cells can be found on tumor-associated fibroblasts infiltrating B16 melanomas [46] but their association with the vasculature has not been investigated. It is likely that in certain malignancies, fibroblasts might be found in the vicinity of the vasculature [47], but this occurs normally in conjunction with alpha-smooth muscle actin expression, conferring these cells a myofibroblast phenotype. In our study, as analyzed by microarray, we did not detect significant differences in alpha smooth muscle actin expression, indicating that the decrease in PDGFRA expression may indeed reflect down-regulation of the B-type subset of pericytes. It is tempting to speculate that the shift towards an increase in type-A pericytes could confer the microvascular environment with features that enhance malignant cell intravasation. Increased expression of *Pten* is, however, likely to have profound effects on endothelial function since this gene reduces phosphatidyl-3’-inositol levels and thus suppresses PI3K and Akt activities. These play major roles for angiogenesis and vascular permeability [48] and thus a reduction in these would be predicted to be deleterious for vascular integrity.

The compromised vasculature in *Shb* deficient mice increases the risk of intravasation of melanoma cells allowing them to disseminate in blood and infiltrate target tissues such as lung. Apparent is the fact that lung seeding of metastases after tail vein injections was not different between the genotypes, further implicating local vascular changes in the primary tumors as responsible for the effects. We were unable to detect increased metastasis to the liver of *Shb* +/− insulinomas [15] and the discrepancy between those findings and the current may lie in the difference in the local angiogenic milieu, which is probably dependent on a multitude of factors in melanomas whereas RIP-Tag2 insulinomas are highly dependent on VEGF and FGF-2 [49].

## Conclusions

Absence of *Shb* promotes B16F10 tumor metastasis due to increased vascular permeability and reduced pericyte/myofibrillar cell coverage of endothelial cells, thus allowing intravasation and vascular dissemination of tumor cells. The data support a model in which tumor metastasis is affected in a context-dependent manner by the absence of *Shb*, contingent on the local angiogenic environment and how it affects vascular permeability and immune cell recruitment. Since Shb is a signaling protein participating in angiogenic responses, this finding has implications for choosing an appropriate strategy for inhibiting tumor expansion by anti-angiogenic treatment without simultaneously increasing tumor metastasis.

## Additional files

Additional file 1: Figure S1. B16F10 melanoma growth (A) and times of tumor resection and animal sacrifice (B). B16F10 melanoma cells (2X10^5^) were injected subcutaneously on day zero on the backs of *Shb* +/+ and +/− mice. Tumor sizes on day 10, 12, 14 and at removal are shown. After tumor removal, mice were maintained for some time until sacrificed, at which numbers of metastases were determined. Times of tumor resection and animal sacrifice are given. Means ± SEM for n = 5 on day 10, n = 24 on day 12 and n = 23 (+/+) and n = 19 (+/−) on day 14. For time of resection and sacrifice, n = 16-21.
Additional file 2: Figure S2. Lung seeding of tail vein-injected B16F10 melanoma cells in *Shb* +/+ and +/− mice. (A) Numbers of lung seeding metastases for each mouse at three weeks after tail vein injections of B16F10 cells (p > 0.77). (B) Numbers of metastases determined as categories (1 = 1-5, 2 = 5-10, 3 > 10). Means ± SEM are given (p > 0.61). Nine mice of each genotype were injected with 200000 cells.
Additional file 3: Table S1. Gene expression in Shb +/- vascular fragments relative wild type. Values are log2 expression wild type, log2 fold change in expression (+/-) and p values for five preparations of vascular fragments from each genotype.

## Abbreviations

VEGF: Vascular endothelial growth factor
VEGFR: VEGF receptor
SHB: Src homology-2 domain protein B
FGF: Fibroblast growth factor
PDGF: Platelet-derived growth factor
VE-cadherin: Vascular endothelial-cadherin
PlGF: Placental growth factor
IL: Interleukin
CCL: Chemokine C-C motif ligand
CCR: CCL receptor
CXCL: Chemokine C-X-C motif ligand
CXCR: CXCL receptor
GM-CSF: Granulocyte macrophage colony-stimulating factor
NOS: Nitric oxide synthase
FAK: Focal adhesion kinase
ERK: Extracellular signal-regulated kinase
RT2: Rat insulin promoter driven SV40 large T antigen-2
