# Supplementary figures and images for: Vascular dysfunction and increased metastasis of B16F10 melanomas in Shb deficient mice as compared with their wild type counterparts
Source: BMC Cancer. 2015 Apr 8;15:234. doi: 10.1186/s12885-015-1269-y (PMC4392795; doi:10.1186/s12885-015-1269-y)

Additional file 1

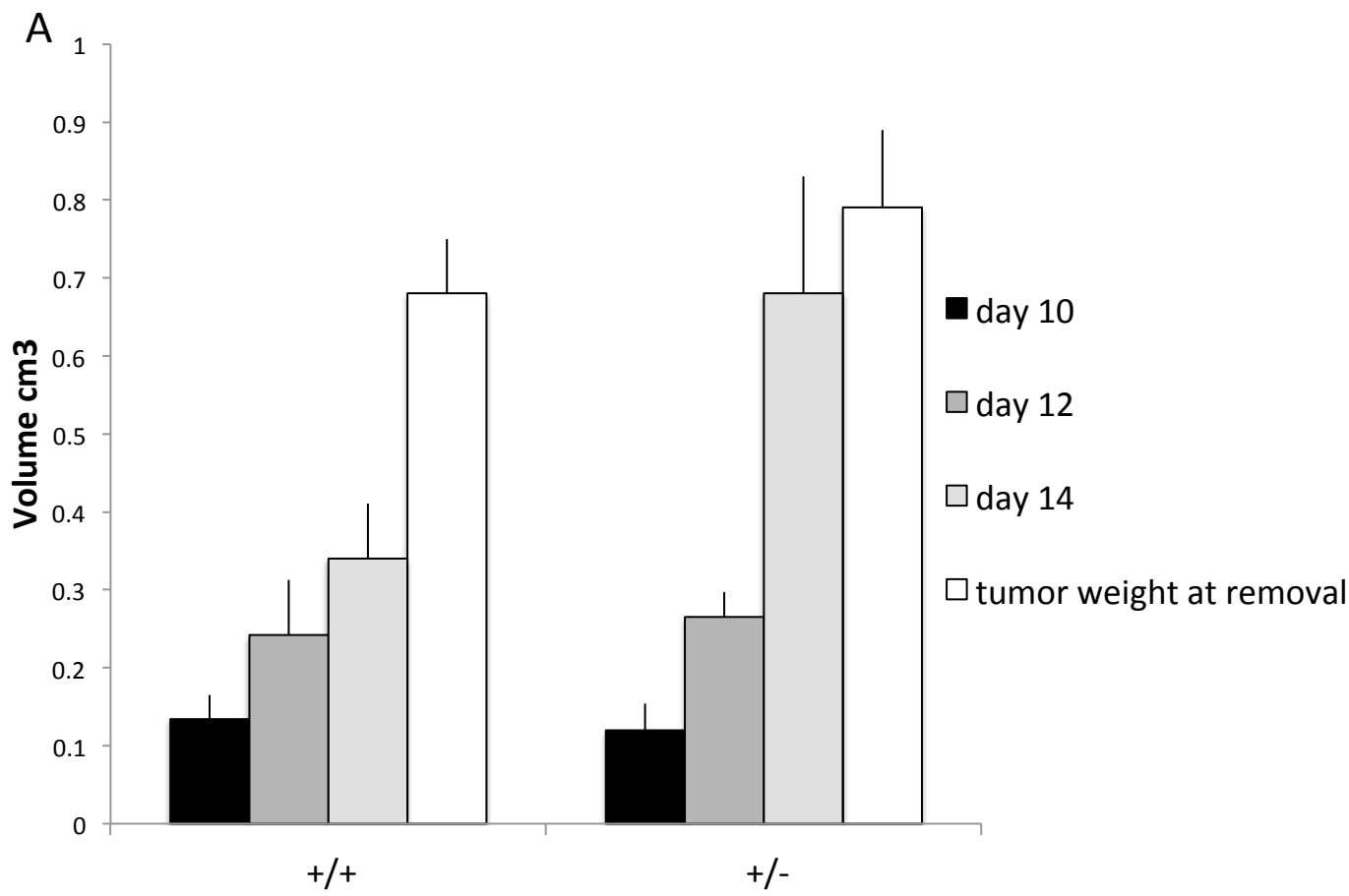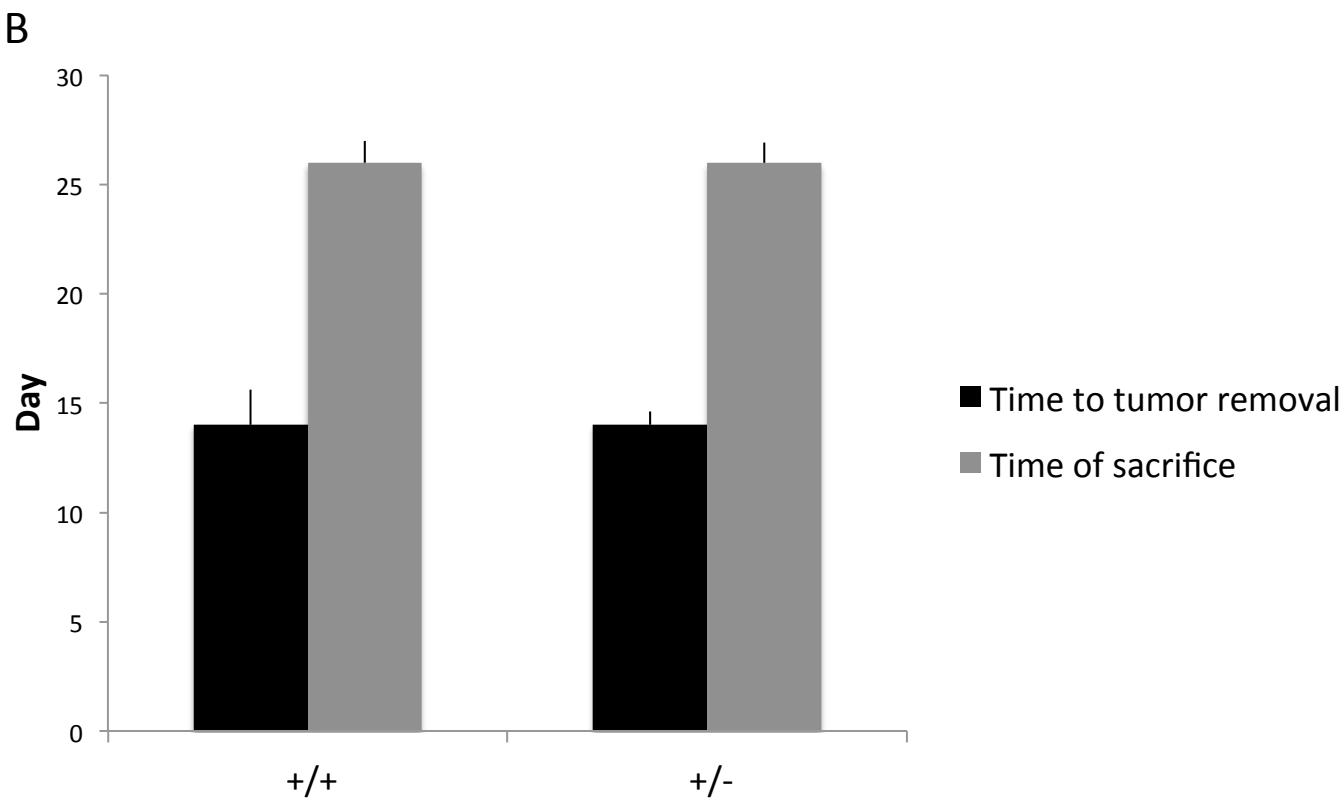

Supplement: Additional file 1: Figure S1. — B16F10 melanoma growth (A) and times of tumor resection and animal sacrifice (B). B16F10 melanoma cells (2X105) were injected subcutaneously on day zero on the backs of Shb +/+ and +/− mice. Tumor sizes on day 10, 12, 14 and at removal are shown. After tumor removal, mice were maintained for some time until sacrificed, at which numbers of metastases were determined. Times of tumor resection and animal sacrifice are given. Means ± SEM for n = 5 on day 10, n = 24 on day 12 and n = 23 (+/+) and n = 19 (+/−) on day 14. For time of resection and sacrifice, n = 16-21. [file 12885_2015_1269_MOESM1_ESM.pdf]

Additional file 2

A

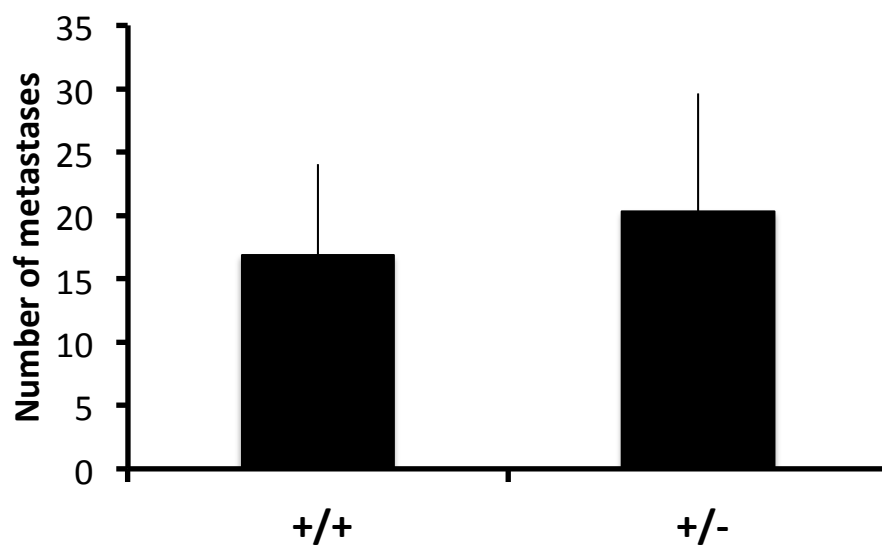

B

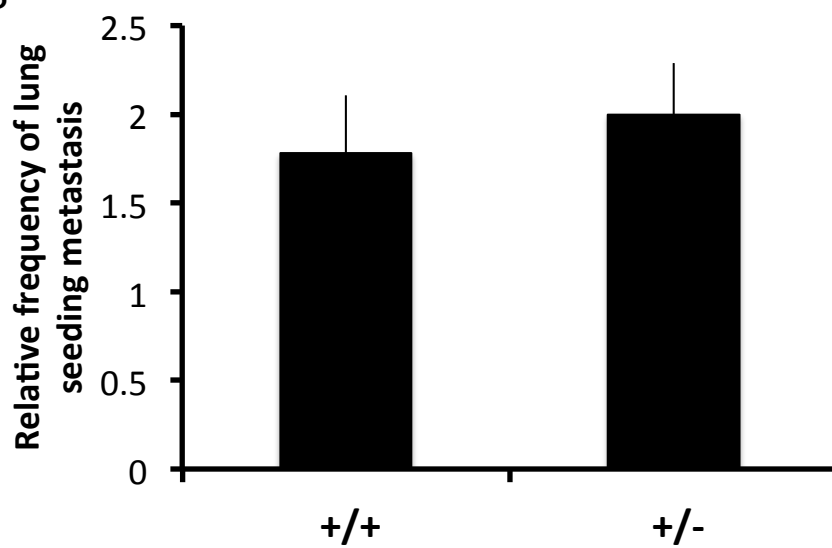

Supplement: Additional file 2: Figure S2. — Lung seeding of tail vein-injected B16F10 melanoma cells in Shb +/+ and +/− mice. (A) Numbers of lung seeding metastases for each mouse at three weeks after tail vein injections of B16F10 cells (p > 0.77). (B) Numbers of metastases determined as categories (1 = 1-5, 2 = 5-10, 3 > 10). Means ± SEM are given (p > 0.61). Nine mice of each genotype were injected with 200000 cells. [file 12885_2015_1269_MOESM2_ESM.pdf]
